# Evaluation of the Vaginal Panel Realtime PCR kit (Vircell, SL) for diagnosing vaginitis: A comparative study with routinely used diagnostics

**DOI:** 10.1371/journal.pone.0313414

**Published:** 2024-11-06

**Authors:** Isabel Amor, Ana Alberola, Adolfo De Salazar, Laura Viñuela, Sara Úbeda-Portugués, María Isabel Galán, Pablo Mendoza, Federico García

**Affiliations:** 1 Department of Research and Development in Molecular Diagnostic, Vircell S.L., Granada, Spain; 2 Department of Biochemistry, Immunology and Molecular Parasitology, Universidad de Granada, Granada, Spain; 3 Department of Clinical Microbiology, Hospital Universitario Clínico San Cecilio, Granada, Spain; 4 Instituto de Investigación Ibs.Granada, Granada, Spain; 5 Ciber de Enfermedades Infecciosas, CiberInfec, Madrid, Spain; GGD Amsterdam, NETHERLANDS, KINGDOM OF THE

## Abstract

Vaginitis is a prevalent clinical disorder associated with several adverse health consequences, prompting women to seek medical care. In this study we evaluate the Vaginal Panel Real-Time PCR kit (qPCR test) against routinely used diagnostics for detection of bacterial vaginosis (BV), vulvovaginal candidiasis (VVC), and trichomoniasis. A total of 1011 vaginal swab specimens were analyzed. The routinely diagnostic methods for BV was Gram stain-based Nugent score. VVC presence was detected by culture, and *Candida* species were identified using MALDI-TOF MS. *Trichomonas vaginalis* was identified by culture in a selective medium. Molecular analyses were conducted on the MagXtract^®^ 3200 System and analyzed using the CFX96^™^ Real-Time PCR Detection System. The sensitivity, specificity, positive predictive value, and negative predictive value of the qPCR test compared to the reference method for BV diagnosis was 93.1%, 88.8%, 90.1% and 92.2%, respectively, with a Kappa value of 0.82. For *Candida* species, sensitivity, specificity, positive predictive value, and negative predictive value were 96.0%, 98.4%, 95.3%, and 98.7%, respectively. The qPCR test detected 32 additional positive samples for *Candida* not reported by the routinely used diagnostics. For trichomoniasis, the qPCR test identified *T*. *vaginalis* in fifteen specimens, despite no microscopic detection in cultured specimens. Our results demonstrate that the Vaginal Panel Real-Time PCR kit shows optimal concordance with routinely used diagnostics for diagnosing vaginitis. Furthermore, enhancing detection of *T*. *vaginalis*. However, further validation studies are necessary to confirm its full diagnostic accuracy. The use of nucleic acid amplification tests (NAATs) provides rapid and accurate diagnosis, crucial for early detection and treatment of vaginitis.

## Introduction

Vaginitis is a prevalent clinical disorder that often presents with symptoms such as burning, odor, itching, and irritation [1,2]. It significantly impacts a large proportion of the female population, leading to frequent consultations in both primary and specialized healthcare settings [3,4]. The most frequently vaginitis associated conditions are bacterial vaginosis (BV), vulvovaginal candidiasis (VVC) and trichomoniasis [2].

BV is a vaginal dysbiosis characterized by a depletion of commensal *Lactobacillus* species and an overgrowth of anaerobic bacteria such as *Gardnerella spp*., *Fannyhessea vaginae* (previously known as *Atopobium vaginae*), *Prevotella*, *Bacteroides* and *Mobiluncus* species in the vaginal microbiota. BV is the most common cause of abnormal vaginal discharge among reproductive-age women [5,6]. The worldwide prevalence of BV in the general population is significant, with rates varying between 23% and 29% across various regions. It presents an estimated incidence of 27% in the United States and 23% in Europe [7]. *Lactobacillus* species play an important role in host defense mechanisms to protect the vaginal microbiome maintaining a balanced and acidic environment. Substances such as lactic acid, hydrogen peroxide and bacteriocins maintain a low vaginal pH inhibiting the growth of opportunistic pathogens [8,9]. VVC is a fungal infection caused by *Candida* species and it ranks as the second most prevalent cause of vaginal infection following BV. It is estimated that 70–75% of women will have at least one episode during their reproductive live [10]. Although the most common and pathogenic species is *Candida albicans* in 90% of the cases, the presence of other species including *C*. *glabrata* (*Nakaseomyces glabrata*), *C*. *tropicalis*, *C*. *parapsilosis* and *C*. *krusei* (*Pichia kudriavzevii*) is emerging [11,12]. Trichomoniasis, caused by the parasitic protozoan *Trichomonas vaginalis*, is the most common nonviral sexually transmitted infection (STI) associated with multiple adverse sexual and reproductive health outcomes in both women and men [13,14]. BV and trichomoniasis are associated with an increased risk of acquiring STIs and can lead to pelvic inflammatory disease, adverse obstetric outcomes, and infertility. Severe or recurrent cases of VVC can also increase susceptibility to STIs and complications in pregnancy [11,13,15]. Several factors might contribute to the development of vaginitis, including age, ethnicity, pregnancy, sexual activity, the use of antibiotics or contraceptives, and hormonal changes [12,14,16]. Treatment for BV, VVC and trichomoniasis typically involves antibiotics or antifungals, but resistance, biofilm formation, and other factors are prompting the development of alternative therapies such as antiseptics, probiotics, or combination treatments [17–19].

Accurate diagnosis of vaginitis remains challenging due to the multiple etiologic agents that could be involved. Clinical diagnosis is historically based on a combination of clinical assessment, microscopic examination and microbiological culture. Amsel criteria has been traditionally used for the diagnosis of BV. However, the gold standard method is Gram stain using the Nugent score [1,2]. Due to the polymicrobial origin of BV, some studies have described different strategies to provide the most accurate diagnosis but the ideal algorithm is still under discussion [6,20–22]. Wet mount microscopy and culture have been traditionally employed for the diagnosis of *Candida* and *T*. *vaginalis* but they have limitations, especially in terms of sensitivity and specificity. As a consequence of the low sensitivity of these techniques for *T*. *vaginalis*, the use of nucleic acid amplification tests (NAATs) is recommended [1,10,23].

Microscopy and culture methods are time-consuming and require trained technicians. In recent years, new molecular technologies, including NAATs and multiplex next-generation sequencing (NGS), have emerged [6]. Techniques such as real-time polymerase chain reaction (qPCR) allow for a rapid and simultaneous detection of a wide range of microorganism. Therefore, accurate diagnosis of vaginitis remains challenging due to the multiple etiologic agents that might be involved.

The Vaginal Panel Realtime PCR Kit (Vircell, SL) is a semiquantitative multiplex real-time PCR test (qPCR test) CE-IVD approved that provides simultaneous detection of *G*. *vaginalis*, *Lactobacillus spp*. (*L*. *crispatus*, *L*. *jensenii*, *L*. *iners* and *L*. *gasseri*), *F*. *vaginae*, *T*. *vaginalis*, *C*. *albicans*, *C*. *glabrata*, *C*. *krusei* and *Candida spp*. (including *C*. *parapsilosis*/*C*. *tropicalis*/*C*. *dubliniensis*) in two reaction tubes. Pre-dispensed mix A and mix B are provided in a PCR strip format. Mix A allows for the detection of *G*. *vaginalis*, *Lactobacillus spp*., *F*. *vaginae* and *T*. *vaginalis*; while mix B targets *C*. *albicans*, *C*. *glabrata*, *C*. *krusei* and *Candida spp*. Moreover, the qPCR test results are automatically analyzed by a software that classifies the sample into BV and normal microbiota. The objective of this study was to assess the diagnostic accuracy of the Vaginal Panel Realtime PCR Kit for the detection of BV, VVC, and trichomoniasis. We compared its performance against routinely used diagnostics in our hospital: Nugent score for BV, culture and MALDI-TOF MS for VVC, and a combination of microscopy and culture for *T*. *vaginalis*.

## Materials and methods

### Clinical specimens

From 27/06/2022 to 27/09/2022, all consecutive vaginal swab samples submitted to the Microbiology Service of the University Hospital San Cecilio (Granada, Spain) from various medical centers in the region were analyzed. Data access and analysis were performed from 20/10/2022 to 18/11/2022. Study participants ranged in age from 14 to 98 years old. No exclusion criteria were applied. The study was designed and conducted in accordance with the principles outlined in the Declaration of Helsinki. The experimental protocols were approved by the local Ethics Committee of Hospital Universitario Clínico San Cecilio in Granada (Spain), with reference number 1517-N-22. Because the testing conducted was deidentified, informed consent was not deemed necessary for this study.

**Fig 1:**
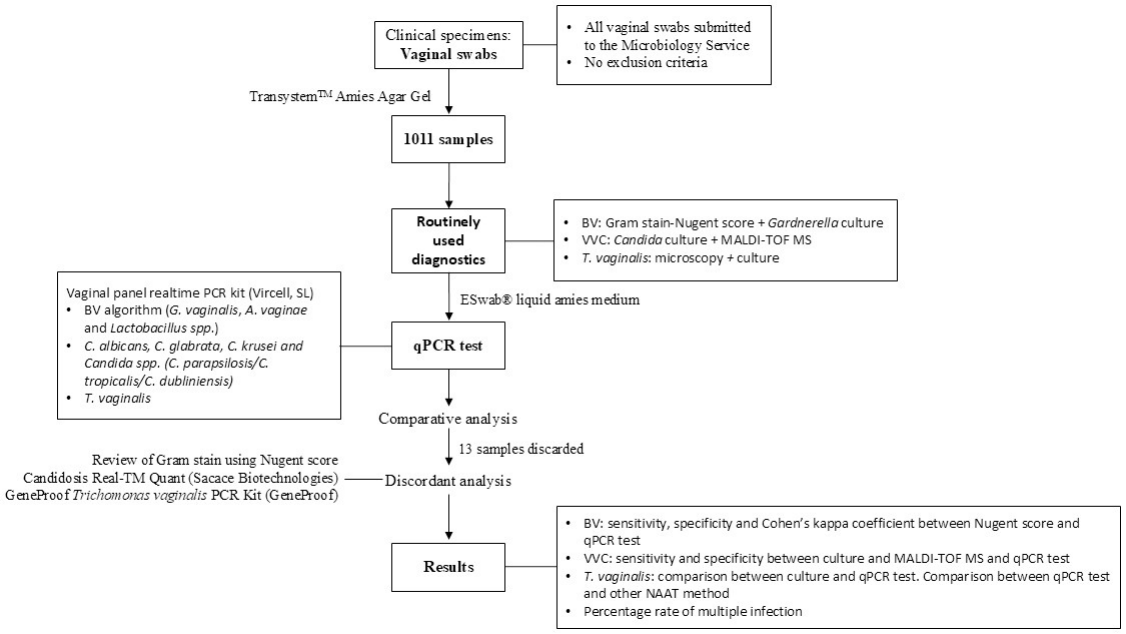
Flowchart illustrates a summary of the experimental methodologies and analytical procedures employed in the study. BV, bacterial vaginosis; VVC, vulvovaginal candidiasis; MALDI-TOF MS, matrix-assisted laser desorption ionization-time of flight mass spectrometry.

One swab was received per patient and collected in Transystem^™^ Amies Agar Gel (Copan Diagnostics Inc., Murrieta, USA). All swabs were subjected to microbiological analysis upon reception and stored at 2–8 °C until qPCR test analysis was performed, in less than 72 h. After routine analysis, resulting data were anonymously collated from all the patients. The study workflow is described in Fig 1, providing an overview of the experimental design, procedures, and data analysis steps performed in our work.

### Routinely used diagnostics

All samples were subjected to microscopic examination as a part of routine diagnostic procedures in the laboratory at the time of the study. For BV diagnosis, vaginal smears were analyzed by Gram strain-based Nugent score. Nugent score evaluates the relative proportions of different bacterial morphotypes to determine the presence and severity of BV. Three types of bacterial morphotypes observed under microscopy are considered to provide a 3-group classification: normal microbiota (score 0–3), intermediate vaginal microbiota (score 4–6) and BV (score 7–10) [24]. In the case of a predominance of Gram-negative, Gram-variable bacilli or clue cells, samples were also plated in *Gardnerella* agar medium (Becton, Dickinson and Company, New Jersey, USA) [25]. For *Candida* diagnosis, all samples were inoculated into CHROMID^®^ Candida agar medium (bioMérieux, Madrid, Spain) at 37 °C for up to 48 h [26]. If culture positive, *Candida* species identification was performed using matrix-assisted laser desorption ionization-time of flight mass spectrometry (MALDI-TOF MS) Maldi Biotyper^®^ System (Bruker Daltonics, Bremen, Germany). Additionally, if leukocytes were observed during the microscopy analysis, the sample was cultured in Roiron medium (Becton Dickinson) at 37 °C for up to 48 h for the diagnosis of *T*. *vaginalis* [27,28].

### Vaginal panel realtime PCR kit (qPCR test)

After routine analysis, each swab was discharged in 1ml of ESwab^®^ liquid amies medium (Copan Diagnostics) for NAAT analysis. Sample processing, nucleic acid extraction and qPCR setup were conducted on a MagXtract^®^ 3200 System (Chroma ATE Inc., Taoyuan, Taiwan) according to the manufacturer’s instructions using 300 μl of sample. TANBead Nucleic Acid Extraction Kit (Taiwan Advanced Nanotech Inc, Taoyuan, Taiwan) was used for nucleic acid purification and isolation, based on nanomagnetic bead technology. An elution volume of 80 μl was obtained and 5 μl was added to each PCR reaction tube (mix A + mix B).

The qPCR assays were carried out using a CFX96^™^ Real-Time PCR Detection System thermal cycler (Bio-Rad Laboratories, Hercules, USA). Negative and positive controls provided in the qPCR test were analyzed in each run to check the amplification process. Vircom middleware (Vircell SL, Granada, Spain) was used for automatic interpretation of results. The qPCR test simultaneously detected 8 microorganisms as described previously. The result analysis software provides assessment of the ratio between the total amount of two bacteria associated with BV (*G*. *vaginalis* and/or *F*. *vaginae*) and the total amount of *Lactobacillus spp*. in the vaginal microbiota for BV diagnosis. Additionally, it provides a four-grade classification based on the quantity of these microorganisms: normal microbiota grade 1 (NM G1), normal microbiota grade 2 (NM G2), BV grade 3 (BV G3) and BV grade 4 (BV G4). NM G1 is reported when only *Lactobacillus spp*. is present. NM G2 is reported if *Lactobacillus spp*. is predominant but *G*. *vaginalis* and/or *F*. *vaginae* are also present. BV G3 is obtained if *Lactobacillus spp*. is present but dominated by *G*. *vaginalis* and/or *F*. *vaginae*. BV G4 is reported in two scenarios: first, in a microbiota associated with BV or abnormal microbiota where *Lactobacillus spp*. is scarce or absent and *G*. *vaginalis* and/or *F*. *vaginae* dominate; second, when none of these three microorganisms are present in the sample. If no bacteria are detected, microbiota alterations may be due to microorganisms not covered by the qPCR kit or other factors affecting the microbiota, such as antibiotic use. The qPCR test included an internal control (endogenous human *RNase P* gene*)* for monitoring the carry-over of amplification inhibitors, sample DNA integrity and the correct amplification set-up.

### Discordant results

In case of discrepancy in the results obtained for *Candida spp*.and *T*. *vaginalis* between routinely diagnostic methods and the qPCR test, a second NAAT method was performed. The Candidiosis Real-TM Quant (Sacace Biotechnologies Srl, Como, Italy) and the GeneProof *Trichomonas vaginalis* PCR Kit (GeneProof as, Brno, Czech Republic), both CE-IVD approved, were used, respectively. For BV, each Gram-stained slide was reviewed by two independent reviewers, both of whom were blinded to the initial interpretation. Agreement between at least two reviewers was required for the final interpretation.

### Data analysis

The agreement rate for BV diagnosis between the routinely used diagnostics and qPCR test was determined using Cohen’s kappa coefficient (κ). A kappa value less than 0.20 indicates poor agreement; a range between 0.21–0.40 indicates fair agreement; 0.41 to 0.60 indicates moderate agreement; 0.61 to 0.80 indicates substantial agreement, and 0.81 to 1.00 indicates almost perfect agreement. Sensitivity, specificity, positive predictive value (PPV), and negative predictive value (NPV) were determined using MedCalc statistical software (version 5.00.017). The McNemar test (MedCalc software) was used to calculate the statistical significance of the BV and VVC analysis results, with a *P* value less than 0.05 considered statistically significant. The correlation between the routine clinical assessment and molecular results was assessed using scatter plots in GraphPad Prism version 8.0.2.

## Results

A total of 1011 vaginal swab specimens were collected for the study. Thirteen samples were excluded from the final analysis: two provided invalid qPCR results, six could not confirm *Candida* results by a third method (described below), and five specimens had unreadable smears. The overall prevalence of each microorganism based on the routine diagnostic assessment compared with the qPCR test is presented in Table 1.

**Table 1:** Overall prevalence of BV, candidiasis and trichomoniasis determined by the routinely used diagnostics and qPCR test.

	Routinely used diagnostic assessment			Vaginal Panel Realtime PCR Kit assessment		
	N. specimens	Prevalence (%)	95% CI	N. specimens	Prevalence (%)	95% CI
BV^a^	421	42.2%	38.17–46.19	435	43.6%	39.51–47.66
**Total Candida**	252	25.3%	22.15–28.35	254	25.5%	22.34–28.57
*C*. *albicans*	221	22.1%	19.24–25.05	219	21.9%	19.05–24.84
*C*. *glabrata*	17	1.7%	0.90–2.51	19	1.9%	1.05–2.76
*C*. *krusei*	4	0.4%	0.01–0.79	4	0.4%	0.01–0.79
*Candida spp*.^b^	5	0.5%	0.06–0.94	7	0.7%	0.18–1.22
Codetection^c^	5	0.5%	0.06–0.94	5	0.5%	0.06–0.94
***T*. *vaginalis***	0	0.0%		15	1.5%	0.75–2.26

^a^BV in the routinely used diagnostics was considered when the Nugent score was 7–10. Samples with intermediate Nugent score (4–6) were not included in the analysis.

^b^The routinely used diagnostic assessment specifically detected *C*. *parapsilosis*.

^c^Codetection refers to dual detection of *Candida* species.

BV, bacterial vaginosis; CI, confidence interval.

### Bacterial vaginosis

The Gram stain-based Nugent score, classified 421 vaginal swabs as BV (score 7 to 10), 384 vaginal swabs as normal microbiota (score 0 to 3), and 193 vaginal swabs as intermediate (score 4 to 6). The qPCR test classified 435 samples as BV and 370 samples as normal microbiota.

We compared the results of both methods for samples classified as BV and normal microbiota by the routinely used diagnostics to assess concordance in BV diagnosis. The sensitivity was 93.1%, and specificity was 88.8%. The differences in sensitivity and specificity did not reach statistical significance (*P* = 0.125). Cohen’s kappa coefficient for BV and normal microbiota were also calculated, yielding a kappa value of 0.82, which indicates almost perfect agreement. A summary of the obtained data is presented in Table 2. Out of 193 specimens characterized as intermediate by routinely used diagnostics, 117 (60.6%) were interpreted as BV, and 76 (39.4%) as normal microbiota by the qPCR test.

**Table 2:** Clinical performance comparation between the Nugent score and qPCR test for detecting BV.

	Vaginal Panel Realtime PCR Kit						
Nugent score	N. specimens positive for BV (G3 and G4)	N. specimens positive for normal microbiota (G1 and G2)	% Sensitivity (95% CI)	% Specificity (95% CI)	% PPV (95% CI)	% NPV (95% CI)	Kappa index (95% CI)
BV^a^	392	29	93,1 (90,3–95,3)	88,8 (85,2–91,8)	90,1 (87,3–92,4)	92,2 (89,2–94,4)	0,82 (0,781–0,860)
**Normal microbiota**	43	341					

^a^BV were considered when the Nugent score was 7–10 and normal microbiota in case of Nugent score 0–3. No growth of *Gardnerella spp*. was found in specimens classified as normal microbiota. However, a high percentage of the specimens classified as BV reported positive growth of this bacterium.

BV, bacterial vaginosis; CI, confidence interval; PPV, positive predictive value; NPV, negative predictive value.

The qPCR test detects and provides semi-quantification of *Lactobacillus spp*., *G*. *vaginalis* and *F*. *vaginae* targets individually. For samples classified as BV, the percentage of those containing both *G*. *vaginalis* and *F*. *vaginae* was compared to the percentage containing only one of these bacteria. The percentage of cases where no bacteria were detected (including *Lactobacillus spp*.), was also determined. The results showed a high percentage of samples contained both *G*. *vaginalis* and *F*. *vaginae* (53.1%), with respect to only one of these bacteria (19.2% and 4.2%, respectively). No bacteria was detected in 23.6% of the samples.

**Fig 2:**
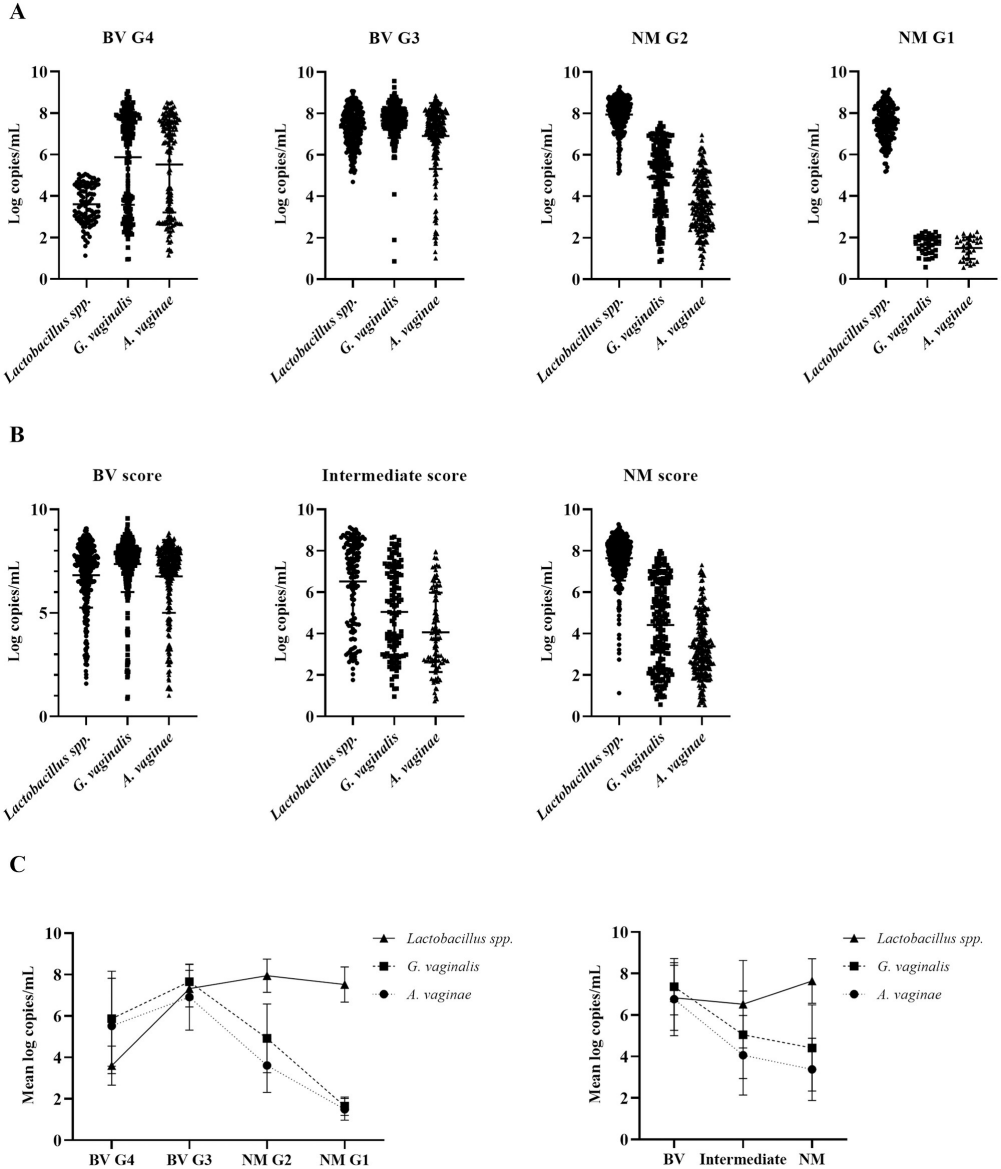
Scatter dot plots show the bacterial load (log copies/mL) of *Lactobacillus spp*., *G*. *vaginalis* and *F*. *vaginae* for the four categories of the Vaginal Panel Realtime PCR Kit **(A)** and the three categories of Nugent score **(B).** The mean and standard deviation (SD) are represented for each microorganism **(C)**. Comparing the profiles of both methods, the qPCR test shows a greater variation in the bacterial load of the three microorganisms between BV G4 and NM G1 **(A)**. In contrast, Nugent score shows a steadier profile across the different scores **(B)**. G1-G4, grade 1, 2, 3 and 4; BV, bacterial vaginosis; NM, normal microbiota.

In addition, further characterization of the specimens was performed for quantitative analysis of the data. The bacteria load of *Lactobacillus spp*., *G*. *vaginalis* and *F*. *vaginae* were determined using standard curve for each bacterium. Bacterial load quantification profiles for each scoring category of Gram stain-based Nugent and qPCR test are shown in Fig 2. The mean and standard deviation (SD) were determined **(**Fig 2C). The qPCR test yielded a gradual profile with a progressive increase in *Lactobacillus spp*. load from BV to normal microbiota ranging from 3.6 to 7.5 log copies/mL. *G*. *vaginalis* and *F*. *vaginae* loads increased slightly from BV G4 to BV G3 (5.9 to 7.7 log copies/mL and 5.5 to 6.9 log copies/mL, respectively) but showed a progressive decrease from BV G3 to NM G1 (7.7 to 1.6 log copies/mL and 6.9 to 1.5 log copies/mL, respectively; Fig 2A and 2C). The routinely used diagnostics demonstrated a similar *Lactobacillus* load profile between BV and normal microbiota (6.8 to 7.6 log copies/mL). For *G*. *vaginalis* and *F*. *vaginae*, the diagnostics showed a gradual decrease (7.4 to 4.4 log copies/mL and 6.8 to 3.4 log copies/mL, respectively; Fig 2B and 2C. Notably, the BV G4 category showed a low *Lactobacillus spp*. load with two distinct populations of *G*. *vaginalis* and/or *F*. *vaginae* based on the qPCR test algorithm. This bimodal distribution is attributed to the presence of two populations within this grade: samples with low and high loads of *G*. *vaginalis* and *F*. *vaginae* bacteria (Fig 2A).

### Vulvovaginal candidiasis

A total of 258 specimens were reported to have the presence of *Candida* species using routinely used diagnostics, with *C*. *albicans* being the most frequently detected species followed by *C*. *glabrata*. Six samples were excluded from the final analysis, as *Candida* presence could not be confirmed by a third method, and the original samples could not be recovered. The sensitivity, specificity, PPV and NPV of the qPCR test compared to the routinely used diagnostics are shown in Table 3. The overall sensitivity for *Candida* was 96.0%, and specificity was 98.4%. The sensitivity and specificity for the different *Candida* species were very similar and showed no statistical significance (*P* > 0.625).

**Table 3:** Clinical performance results for *Candida* species. Culture and MALDI-TOF MS were compared with qPCR test.

	Vaginal Panel Realtime PCR Kit							
Culture and MALDI-TOF MS	True positive	False positive	True negative	False negative	% Sensitivity (95% CI)	% Specificity (95% CI)	% PPV (95% CI)	% NPV (95% CI)
***C*. *albicans* (N = 221)**	212	7	770	9	95.9 (92.4–98.1)	99.1 (98.2–99.6)	96.8 (93.5–98.5)	98.8 (97.8–99.4)
***C*. *glabrata* (N = 17)**	17	2	979	0	100 (80.5–100)	99.8 (99.3–100)	89.5 (68.0–97.1)	100
***C*. *krusei* (N = 4)**	4	0	994	0	100 (39.8–100)	100 (99.6–100)	100	100
***Candida spp*. (N = 5)**	4	3	990	1	80.0 (28.4–99.5)	99.7 (99.1–99.9)	57.1 (28.4–81.8)	99.9 (99.4–100)
**Codetection** ^a^ **(N = 5)**	5	0	993	0	100 (54.1–100)	100 (99.6–100)	100	100
**Candida total (N = 252)**	242	12	734	10	96.0 (92.8–98.1)	98.4 (97.2–99.2)	95.3 (92.0–97.3)	98.7 (97.6–99.3)

^a^ Codetection refers to dual detection of *Candida*. In five specimens, both methods reported a positive result for *C*. *albicans* and *C*. *glabrata*.

CI, confidence interval; PPV, positive predictive value; NPV, negative predictive value.

Additionally, the qPCR kit detected 32 positive samples for *Candida* that were not reported by the routinely used diagnostics. These positive results were confirmed by testing with another NAAT method, the Candidosis Real-TM Quant. Among the additional positive samples detected by the qPCR kit, 23 specimens tested positive for *C*. *albicans*, 4 specimens for *C*. *glabrata*, 1 specimen for *Candida spp*., and 4 specimens showed codetection, indicating the presence of a combination of two species.

### Trichomoniasis

The microscopy examination revealed 57 samples with the presence of leukocytes, presumptively positive for *T*. *vaginalis*. Following laboratory procedures, these samples were cultured in Roiron medium, but the parasite was not microscopically observed in any of the cultured specimens. The Vaginal Panel Realtime PCR kit detected fifteen *T*. *vaginalis* positive samples among the entire samples analyzed. To confirm the result obtained with the qPCR test, these fifteen positive samples were tested by another NAAT method, the GeneProof *Trichomonas vaginalis* PCR Kit. The alternative molecular method yielded a positive result for *T*. *vaginalis* in twelve of these specimens.

### Multiple infection

**Fig 3:**
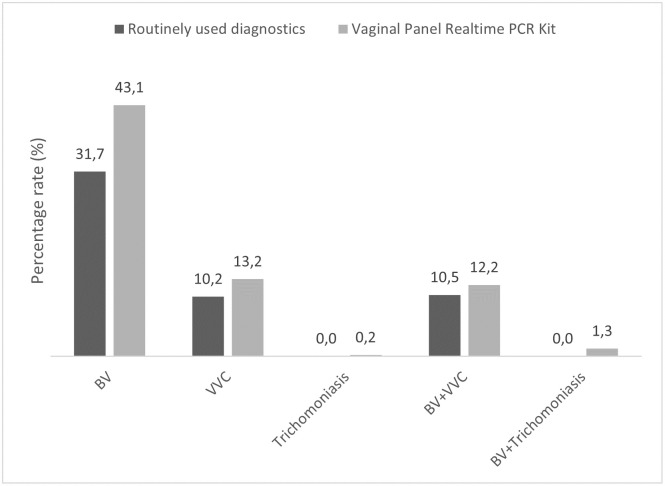
Percentage rate of samples with single and multiple infection reported by routinely used diagnostics and the Vaginal panel realtime PCR Kit. BV, bacterial vaginosis; VVC, vulvovaginal candidiasis.

Vaginitis disorder might be due to single or multiple causes. A combined analysis of the results was also performed. The percentage rate of positive samples for BV, VVC and trichomoniasis in single and multiple infection were also determined (Fig 3). The routinely used diagnostics identified more cases of multiple infection of BV with VVC (10.5%) that single VVC infection (10.2%). In case of trichomoniasis, high rate of samples presented multiple infection with BV (0.2% vs. 1.3%).

## Discussion

The vaginal microbiome plays a crucial role in women’s health. Alterations in the vaginal microbiome can lead to vaginal infections, such as BV, VVC and trichomoniasis, among others. Early diagnosis and treatment are likely to reduce the risk of complications and recurrences. This study provides a comparison of the Vaginal Panel Realtime PCR Kit (Vircell SL) against routinely used diagnostics for diagnosing the primary causes of vaginitis.

Clinical assessment of vaginitis typically relies on a combination of factors, including clinical symptoms, medical history, microscopy techniques, and microbiological culture [1,2]. While some studies assert that *Gardnerella spp*. is the predominant bacteria responsible for BV and serves as a good predictor of infection, it is important to note that these bacteria can also be present in women without BV. Additionally, other microorganisms may also be involved [6,16,20]. Specifically, the combination of *G*. *vaginalis* with *F*. *vaginae* has been strongly linked to biofilm formation, contributing to recurrence and therapeutic challenges [4,6,18,29,30]. Given the varied etiologies and bacterial profiles involved, the diagnostic algorithm for BV remains a topic of ongoing discussion.

In the case of BV, Nugent score categorize samples into three groups, whereas the Vaginal Panel Realtime PCR Kit classifies them into two groups, with each group further divided into two grades. Because of this difference in classification, specimens identified as intermediate by Nugent were excluded from the result analysis shown in Table 2. Previous studies assessing molecular methods also encountered the necessity to exclude these samples due to disparities in classification between traditional and molecular methods [31,32]. The results demonstrated very good performance of the qPCR test, indicating that the targets and algorithm included are reliable predictors for BV diagnosis.

Biofilms formation plays a crucial role in the pathogenesis of BV, recurrences, and treatment failure [30]. The results obtained with the qPCR test revealed a high percentage of samples positive for both *G*. *vaginalis* and *F*. *vaginae*, compared to samples positive for only one of these bacteria. The Gram stain-Nugent score method does not enable the identification of *F*. *vaginae*, potentially leading to underdiagnosis of this pathogen. Therefore, the inclusion of *F*. *vaginae* in the qPCR test is essential for making informed treatment choices [20,33].

The quantitative analysis of bacterial load using standard curves revealed distinct patterns across Nugent scoring categories and qPCR test results. While routine diagnostic methods showed a similar *Lactobacillus* load profile across the three categories, the qPCR test demonstrated a gradual increase in *Lactobacillus* load from BV G4 to NM G1 (Fig 2C). The qPCR test ability to detect and quantify these bacterial species provides a more precise assessment of BV and the overall health of the vaginal microbiota. Notably, the qPCR test results identified a bimodal distribution within the BV G4 category, suggesting that not all BV G4 cases are identical. Some may represent transitional states of microbial balance, with varying levels of *G*. *vaginalis* and *F*. *vaginae*. This distinction is clinically important, as patients in the BV G4 category may benefit from different therapeutic strategies based on their bacterial load profiles. In contrast, routinely diagnostic methods showed similar *Lactobacillus* spp. loads between BV and normal microbiota, but they demonstrated a more consistent decrease in *G*. *vaginalis* and *F*. *vaginae* loads, without the bimodal distribution observed in the qPCR results. This highlights the added value of the qPCR test in detecting subtle variations in bacterial populations.

The vaginal microbiome undergoes fluctuations throughout women’s lives, often in response to hormonal changes [8]. Studies utilizing in-house or commercial NAATs have highlighted the advantages of including *Lactobacillus* as a positive marker of a healthy microbiota [22,34–37]. A meta-analysis conducted by Muñoz-Barreno et al. in 2021 demonstrated that combined therapies involving antibiotics and probiotics enhance cure rates and reduce recurrent infections [17]. Our findings align with previous research, indicating that the inclusion of *Lactobacillus* in BV diagnosis serves as a reliable predictor of infection. We identified 23.6% of BV cases characterized by either the absence or very low levels of anaerobic bacteria alongside *Lactobacillus*. Therefore, its incorporation proves valuable in assessing the state of the vaginal microbiota and identifying cases where infection may be attributable to other microorganisms.

In our study, the sensitivity, specificity, PPV, and NPV for the diagnosis of *Candida* species demonstrated very good concordance between the qPCR test and the routinely used diagnostics (Table 3). The prevalence of *C*. *albicans* was notably higher at 87.7% compared to non-*albicans Candida* species (10.3%), consistent with previously described data [11,12]. However, giving the rising incidence of non-*albicans Candida* species in recent years [11,38], their diagnosis warrants consideration, as they may contribute to recurrent infections and treatment failures [2,11,39]. Fluconazole, commonly prescribed for VVC infections, is effective against many *Candida* species, particularly *C*. *albicans*. However, species such as *C*. *glabrata* and *C*. *krusei* exhibit reduced susceptibility or resistance to this drug, posing treatment challenges [40]. Furthermore, VVC caused by *C*. *glabrata* infection has been associated with a higher risk of recurrence compared to VVC caused by *C*. *albicans* [41]. The qPCR test detects the most common non-*albicans Candida* species, enabling early detection compared to routinely used diagnostics. The results obtained with the qPCR kit revealed 32 additional positive samples for *Candida* that were not identified by routine diagnostic methods. These results were confirmed by another NAAT, Candidosis Real-TM Quant, highlighting the superior capability of NAATs to detect *Candida* infections that may have gone unnoticed with conventional approaches. The codetection observed in 4 samples is also significant, as it indicates mixed infections that may require a different therapeutic approach compared to a mono-species infection. The NAAT ability to identify these combinations can provide valuable information for clinical management, as mixed infections may present additional challenges in terms of treatment and management.

The Vaginal Panel Realtime PCR Kit identified 15 positive cases that were not detected by routinely used diagnostics, microscopy and culture, indicating relatively low specificity. Upon comparing the results from the two NAAT tests, we found very similar outcomes: twelve cases were detected by both methods, while three cases were not confirmed by the other NAAT method, GeneProof *Trichomonas vaginalis* PCR Kit. It remains unclear whether the three positive cases reported by the qPCR test but not confirmed by a third method were false positives or a result of differences in sensitivity between the tests. These results suggest that NAATs provided more reliable results for detecting the parasite than Gram stain and culture. Our findings indicated that detecting *T*. *vaginalis* in clinical samples using traditional diagnostic methods can be challenging due to various factors, including the lower parasite viability and a slow growth rate. This highlights the importance of recommending NAATs for more reliable detection of the parasite [14,27].

Multiple infections involving BV, VVC, and trichomoniasis can occur but are less common than single infections. Traditionally, these infections are considered individually due to their distinct etiology and clinical symptoms. However, the vaginal dysbiosis resulting from BV may predispose the vaginal environment to colonization by other pathogens, increasing the risk of STIs as well as other vaginal infections like VVC and trichomoniasis. In fact, trichomoniasis often drives the microbiome towards BV [13,42]. Multiple infections may lead to more severe symptoms and recurrent episodes, highlighting the importance of accurate diagnosis and appropriate treatment. Our results showed a high percentage rate of multiple BV infection with VVC and BV with trichomoniasis (Fig 3). Understanding the relationship between vaginal dysbiosis due to BV and the increased risk of pathogen colonization is crucial for clinicians to develop effective prevention and treatment strategies, ultimately improving vaginal health and reducing the risk of associated complications and infections.

Despite the results obtained, this study had four main limitations. First, a direct comparison of BV diagnosis could not be established due to the different classifications of vaginal microbiota between methods, as previously described. Second, only one vaginal swab was collected and retested after standard diagnostics. This limited sampling may have affected the detection rate of the NAAT methods. Third, for *T*. *vaginalis*, only discordant samples were analyzed using the GeneProof *Trichomonas vaginalis* PCR Kit. A more comprehensive analysis could involve testing all samples with the alternative PCR method. Additionally, the low rate of positive results was due to the low prevalence of the parasite in our population. Despite this, our results are consistent with those reported by other studies conducted in Spain [43,44]. A better evaluation of this qPCR kit would be to assess it in populations with a higher prevalence of *T*. *vaginalis*. Finally, the anonymity of the participants in the study limited the access to information about symptoms, ethnicity, contraceptive use, STI or menopausal state. All these factors are closely related and have a significant influence on vaginal microbiome variations [2,5,11,13]. This lack of data could limit the ability to analyze potential correlations between these factors and vaginitis diagnosis, potentially affecting the generalizability of the findings. The qPCR test algorithm for BV diagnosis classifies samples as either normal microbiota or BV, eliminating the ’ambiguous’ intermediate scoring of the Nugent criteria. However, we cannot correlate the results with the clinical data to confirm the accuracy of the test results. A deeper insight into clinical data would have allowed for a more meaningful analysis of the test results.

Accurate vaginitis diagnosis is needed to provide better treatment and reduce recurrent infections. Gram stain and wet mount are observer-dependent, require skilled laboratory technicians to identify microorganisms and might generate results complicate to interpret such as the intermediate category in Nugent score. Despite these disadvantages, Gram-stained vaginal smears remain widely used due to its simplicity and cost-effectiveness. However, the implementation of NAATs has increased in the recent years. NAATs allow for the simultaneous detection of several microorganisms and are able to identify bacteria difficult to culture. While traditional laboratory diagnosis takes at least 2–3 days to provide preliminary results, the qPCR test could be available within 3–4 hours. The use of automatic platforms for nucleic acid extraction and PCR setup simplifies and reduces the hands-on time, and may standardize results among laboratories. On the other hand, the higher sensitivity of NAATs might lead to overdiagnosis. A PCR diagnosis independent of smear and culture could increase overall costs. Therefore, it is advisable to combine data generated from these tests with the symptoms and medical history of the patient.

Due to the limitations of conventional diagnostic methods, molecular tests similar to the one evaluated in this study have been developed in recent years to detect the primary causes of vaginitis. These tests are designed to improve accuracy and simplify the current diagnostic procedure. Previous studies have clinically evaluated panels such as the BD MAX^™^ Vaginal Panel (Becton, Dickinson and Company) and Seegene Allplex^™^ Vaginitis (Seegene Inc., Seoul, South Korea) against reference methods, yielding similar results and conclusions to ours [31,37,43]. However, these tests require high-cost automated platforms, which are not always accessible to all laboratories. Furthermore, due to the complexities in diagnosing BV, the ideal algorithm remains undefined. Therefore, it is crucial to evaluate which method is most appropriate for each specific laboratory setting. New insights into the ecology of the vaginal bacterial are obtained with metagenomics and metatranscriptomic techniques. However, these are not tools yet used in the routine clinical microbiology laboratory.

## Conclusions

This study highlights the potential benefits of NAATs for the microbiological diagnosis of vaginitis infections. NAATs offer high sensitivity and specificity, allowing for a more accurate detection of pathogens like *T*. *vaginalis*, *Gardnerella spp*., and *F*. *vaginae*, providing results comparable to traditional methods for detecting BV and improving the detection of trichomoniasis and VVC. Additionally, our results underscore the capability of qPCR test to detect mixed infections, which may require different therapeutic approaches. The ability to deliver results within 3–4 hours significantly improves the speed of diagnosis, facilitating timely treatment and potentially reducing complications and recurrences.

The Vaginal Panel Real-Time PCR kit is an easy tool for diagnosing vaginitis that demonstrated strong performance, yielding unbiased results compared to the routinely used diagnostics methodology and improving the detection rate for *T*. *vaginalis*.
